# Antidepressants cheer up hepatic B1 B cells: Hope for the treatment of autoimmune liver diseases?

**DOI:** 10.3389/fimmu.2022.1083173

**Published:** 2023-01-17

**Authors:** Timm Amendt, Victor L. J. Tybulewicz

**Affiliations:** ^1^ Institute of Immunology, Ulm University, Ulm, Germany; ^2^ The Francis Crick Institute, London, United Kingdom

**Keywords:** B cells, mirtazapine, autoimmune liver diseases, cytokines, natural IgM

## Introduction

Depression is a common mental health disorder affecting over five percent of adults worldwide reducing their health-related quality of life (1). Patients suffering from chronic diseases (e.g. chronic liver diseases, *CLD*) show a drastically increased prevalence of depression that is often treated with antidepressants (2, 3). Interestingly, various antidepressants have been shown to bind to receptors expressed by immune cells (4), thus suggesting immunomodulatory effects. Notably, altered plasma cytokine levels or altered immune cell proliferation have been observed in patients treated with antidepressants (5–7), but little is known about underlying molecular mechanisms. Thus, it is conceivable that antidepressants affect the quality of adaptive immune responses which are critical for protection from infections and effectiveness of vaccines (8, 9).

A central part of adaptive immunity is formed by B cells. In particular, canonical B2 B cells reside in lymphoid organs as follicular (FO) or marginal zone (MZ) B cells co-expressing IgD-class and IgM-class B cell antigen-receptors (BCR). Upon encountering antigen with the BCR, B cells get activated and eventually differentiate into antibody-secreting plasma cells or memory B cells (MBC) (10). Individuals lacking B cells completely show agammaglobulinemia resulting in a severe immunodeficiency which highlights the importance of B cells in adaptive immunity (11).

In addition to canonical B2 B cells, mice also harbor B1 B cells (10) colonizing the peritoneal cavity, liver and other organs (12, 13). Belonging to the class of innate lymphocytes, self-renewing B1 B cells are capable of secreting anti-inflammatory cytokines such as IL-10 (14) and presenting antigens *via* MHC-II molecules (15). However, the most prominent characteristic of B1 B cells is the production of germline-encoded natural IgM autoantibodies (nIgM) (16–20). Critical features of nIgM include low antigen-binding affinity and strong polyreactivity enabling nIgM antibodies to bind and neutralize harmful self-molecules such as oxidized lipids (oxLDL) or advanced glycation end products (AGE) (19–21). Recently, nIgM binding to proinflammatory cytokines such as TNFα or IL1-β has been reported, suggesting a role of nIgM in cytokine clearance and thus cytokine homeostasis (22, 23).

The importance of nIgM for physiological homeostasis was demonstrated by studies showing that mice lacking secreted IgM develop various inflammatory diseases such as atherosclerosis or lupus-like syndrome (17–19). In particular, certain specificities of nIgM antibodies are now being tested in clinical trials since they have been demonstrated to be beneficial in thrombosis (24). Thus, understanding the biology of B1 B cells and corresponding human cells in both health and disease is critical to advance therapies of various diseases.

Besides localizing in classical lymphoid organs such as the spleen or lymph nodes, B cells are also found in the liver (10). Recent studies suggested that the liver can play a critical role in fighting infections by being a generative site for B cell responses (25). However, dysregulation of hepatic B cells might lead to the development of autoimmune liver diseases (AILD) (26, 27) including three different conditions that are accompanied by B cell-mediated pathology: autoimmune hepatitis (AIH), primary biliary cholangitis (PBC) and primary sclerosing cholangitis (PSC).

Historically, AIH has been thought to be a T cell-driven disease associated with impaired regulatory T cell function and autoreactive T cells (28, 29) linked to HLA class II associations (30, 31). Thus, T cells are likely to be important for disease onset. However, while the evidence for direct autoantibody-mediated pathology in AIH is debatable, B cells have been shown to play an important role in pathogenesis (26, 32). A growing body of evidence suggests that B cells contribute crucially to AIH pathogenesis through antigen presentation and cytokine production, thereby regulating T cell responses (32). In particular, AIH mouse model data support this concept by showing increased numbers of IFN-γ and TNF-α-secreting B cells in spleens and livers, highlighting the importance of B cells in AIH pathogenesis (33, 34). Interestingly, preliminary studies of B cell depletion therapy in AIH patients suggest that this may be an effective treatment, supporting the view that B cells participate in AIH pathology (26, 35–38).

PBC is associated with the presence of anti-mitochondrial antibodies (AMA) resulting in inflammation and progressive fibrosis (26, 39). Whereas PSC is a rare disease characterized by fibrosis of the intrahepatic biliary tree resulting in liver cirrhosis (40). Dysregulated microbiota and abnormal antibody responses to commensal bacteria have been proposed as mechanisms of PSC pathogenesis (41).

Taken together, these results show that autoantibodies, antigen presentation and cytokine production by B cells may contribute significantly to pathogenesis and disease progression in AILDs (26, 27). Thus, drugs targeting liver-localized B cells could improve AILD therapy.

This article will discuss recent findings on the effects of mirtazapine on liver-localized B cell biology in light of the current understanding of AILD.

## Mirtazapine enriches B1 B cells in the liver: A beneficial role for hepatic nIgM?

The atypical antidepressant, mirtazapine, shows antagonistic effects on several receptors including histamine receptor H1 (42) and serotonin receptors 5HT3 and 5HT3A (43) expressed by B cells. Almishri and colleagues (44) reported that mice treated with mirtazapine showed a drastic reduction of overall B cell numbers in the liver. In particular, remaining liver-localized B cells were skewed towards B1 B cells. The experiments show that mirtazapine causes increased sinusoidal blood flow which could wash out the more migratory B2 B cells, whilst retaining more slowly migrating B1 B cells.

Since a major feature of B1 B cells is the production of nIgM, it is tempting to speculate that accumulation of hepatic B1 B cells in mice treated with mirtazapine impacts natural autoantibody levels in the liver (45, 46). It would be interesting to examine if animals treated mirtazapine show elevated hepatic nIgM titers (46), or if hepatic B1 B cell accumulation affects certain nIgM specificities possibly involved in regulation of inflammation.

Recently, several studies highlighted the importance of hepatic nIgM for limiting liver autoinflammation and enhancing liver regeneration (45, 47, 48). It has been demonstrated that certain nIgM specificities recognizing Annexin IV (nIgM B4) and subsets of phospholipids (nIgM C2) are capable of promoting liver regeneration post injury (47). Further, liver-localized B1 B cells have been shown to produce a set of nIgM autoantibodies binding to oxidation-specific epitopes. Interestingly, loss of these anti-oxidation nIgMs lead to the development of non-alcoholic fatty liver disease (NALFD) (48). Another study observed that restoration of nIgM production by hepatic B1 B cells drastically improved liver regeneration post injury (45). Together, hepatic nIgMs facilitate rapid removal of apoptotic hepatocytes and neutralize harmful oxidized molecules thereby preventing autoinflammation.

Thus, it is possible that mirtazapine-mediated shifting towards B1 B cells in the liver may lead to to elevated hepatic nIgM titers which could exert liver-protective effects. This preventive protection could be of particular interest in scenarios where patients suffer from low-grade liver inflammation as in obesity (49). Consequently, elevated hepatic nIgM could prevent manifestation of liver autoinflammation and thereby prevent liver injury.

Additionally, it has been shown that a mouse model of PBC is driven by dysregulated B1a B cells (50). In particular, B1a B cells of PBC mice showed significantly lower proliferation rates and downregulation of IL-10 production indicating a loss of regulatory functions. Thus, a diminished B1 B cell compartment could promote autoantibody production and autoreactive T cell recruitment to the liver as observed in PBC mouse models and this might be reversed by mirtazapine treatment.

In summary, Almishri and colleagues (44) have shown that treatment of mice with mirtazapine leads to shifting towards B1 B cells in the liver. It is conceivable that this might elevate hepatic nIgM titers exerting positive effects on liver immune homeostasis.

## Suppression of B cell-derived pro-inflammatory cytokine production by mirtazapine could target AILD at a critical point

Various CLD and AILD are associated with dysregulation of cytokine levels, in particular showing elevated pro-inflammatory cytokine levels that promote autoinflammation (26, 27). It has been reported that hepatic B cells contribute significantly to liver inflammation *via* cytokine production (49). Thus, modulation of B cell-derived hepatic cytokine levels could be a critical step towards curative AILD therapy.

Almishri et al. (44) observed that hepatic B cells of mice treated with mirtazapine show altered cytokine secretion profiles compared to hepatic B cells isolated from control mice. The authors stimulated the isolated hepatic B cells with PMA/ionomycin (51) to determine which cytokines the cells were primed to secrete. Interestingly, upon PMA/ionomycin stimulation, hepatic B cells post-mirtazapine treatment secreted significantly less pro-inflammatory cytokines (IFN-γ, TNF-α, IL-6) compared to controls. Furthermore, secretion of the anti-inflammatory cytokine IL-4, but not IL-10, was increased post *in vitro* stimulation. These data are in agreement with previous work of the group showing effects of mirtazapine treatment on liver immunity and suppression of liver inflammation (52).

A strong association of pro-inflammatory cytokines with AILD pathogenesis was demonstrated in mouse models of AIH. In particular, AIH models revealed increased numbers of IFN-γ and TNF-α producing B cells promoting inflammation (33). Additionally, the second-line treatment, 6-mercaptopurine, showed great effectiveness due to inhibition of IL-6 production in B cells thereby limiting IL-6-mediated activation of pathogenic T_H_1 and T_H_17 cells (32, 53, 54). Notably, mirtazapine treatment could also reduce B cell-derived IL-6 secretion (44).

Further, numbers of IL-10-producing B1 B cells were drastically diminished in AIH mouse models, suggesting the importance of B1 B cells in liver (inflammation) homeostasis. This suggests that a decreased ratio of hepatic B1 to B2 B cells may promote AIH. Treatment of AIH by B cell depletion in mouse models (33) or Rituximab administration in human AIH (36) significantly improved AIH potentially by blocking autoantibody production and B cell-derived cytokine secretion. Further studies support this view by showing that treatment of AIH patients with Rituximab or Belimumab (BAFF inhibitor) are effective in treating the condition (37, 38). In contrast, recent evidence also shows that B cell depletion by Rituximab in experimental murine AIH reduces B cell numbers and IgG levels, but does not improve pathology indicating a discrepancy between human AIH and certain experimental mouse models (55). Furthermore, B cell depletion leads to a systemic immunosuppression which may abolish any protective effect of hepatic B1 B cells. Here, mirtazapine-like drugs could be an elegant therapeutic option to be tested, since mirtazapine lowers pro-inflammatory. Here, mirtazapine-like drugs could be an elegant therapeutic option to be tested since it lowers pro-inflammatory cytokine production whilst enriching for B1 B cells. Thus, it will be interesting to test if treatment of AIH mice with mirtazapine can stop disease progression by enhancing the anti-inflammatory functions of B1 B cells, whilst suppressing B cell-derived pro-inflammatory cytokine secretion thereby limiting T cell responses. Drugs inducing similar effects on hepatic B cells without exerting neurological effects could be an interesting treatment option to test. Particularly, since classical B cell depletion also abolishes all anti-inflammatory effects of B cells, which might be enhanced upon mirtazapine treatment.

Another AILD associated with dysregulation in B cell biology is PBC. Patients suffering from PBC show enlarged B cell compartments in the liver (26). Regarding cytokine profiles, stimulation of PBC-derived B cells with CD40L induces increased secretion of IFN-γ, TNF-α and IL-6 pointing towards an important role of B cell-derived cytokines in PBC progression (56). Further, PBC patients show elevated serum levels of B cell activating factor (BAFF), a cytokine that promotes survival of B cells. Interestingly, levels of BAFF correlate with increased levels of liver enzymes in the serum that indicate liver tissue damage, supporting the hypothesis that liver disease is promoted by B cell-driven autoinflammation (57, 58).

Elevated BAFF levels might be crucially involved in the pathogenesis of AILD since mice overexpressing BAFF spontaneously develop autoimmune diseases due to drastically increased (autoreactive) B cell survival (59, 60). In the case of PBC, it will be interesting to examine if BAFF is secreted by liver-localized cells and if mirtazapine treated mice show decreased BAFF serum levels. Moreover, BAFF was demonstrated to reduce the production of IL-10 and TGF-β by mouse B1 B cells further accelerating autoinflammation in combination with elevated pro-inflammatory cytokine titers (57, 58). These findings taken together with results reported in Almishri et al., suggest that mirtazapine treatment could dampen the effects of BAFF by suppressing secretion of pro-inflammatory cytokines whilst increasing the secretion of anti-inflammatory cytokines.

In sum, AILD such as AIH and PBC are known to involve B cell-derived cytokine dysregulation in pathogenesis showing the need for profile-modulating drugs. Interestingly, mirtazapine is capable of altering the hepatic cytokine profile towards anti-inflammatory molecules and thus it will be interesting to examine effects of mirtazapine treatment in AILD mouse models.

## Discussion

Accumulating evidence suggests a pivotal role of autoantibodies, pro-inflammatory cytokines and dysregulated B1 B cells in the pathogenesis of AILD (26). Thus, understanding the biology of hepatic B cells is crucial for developing novel treatment strategies.

**Figure 1:**
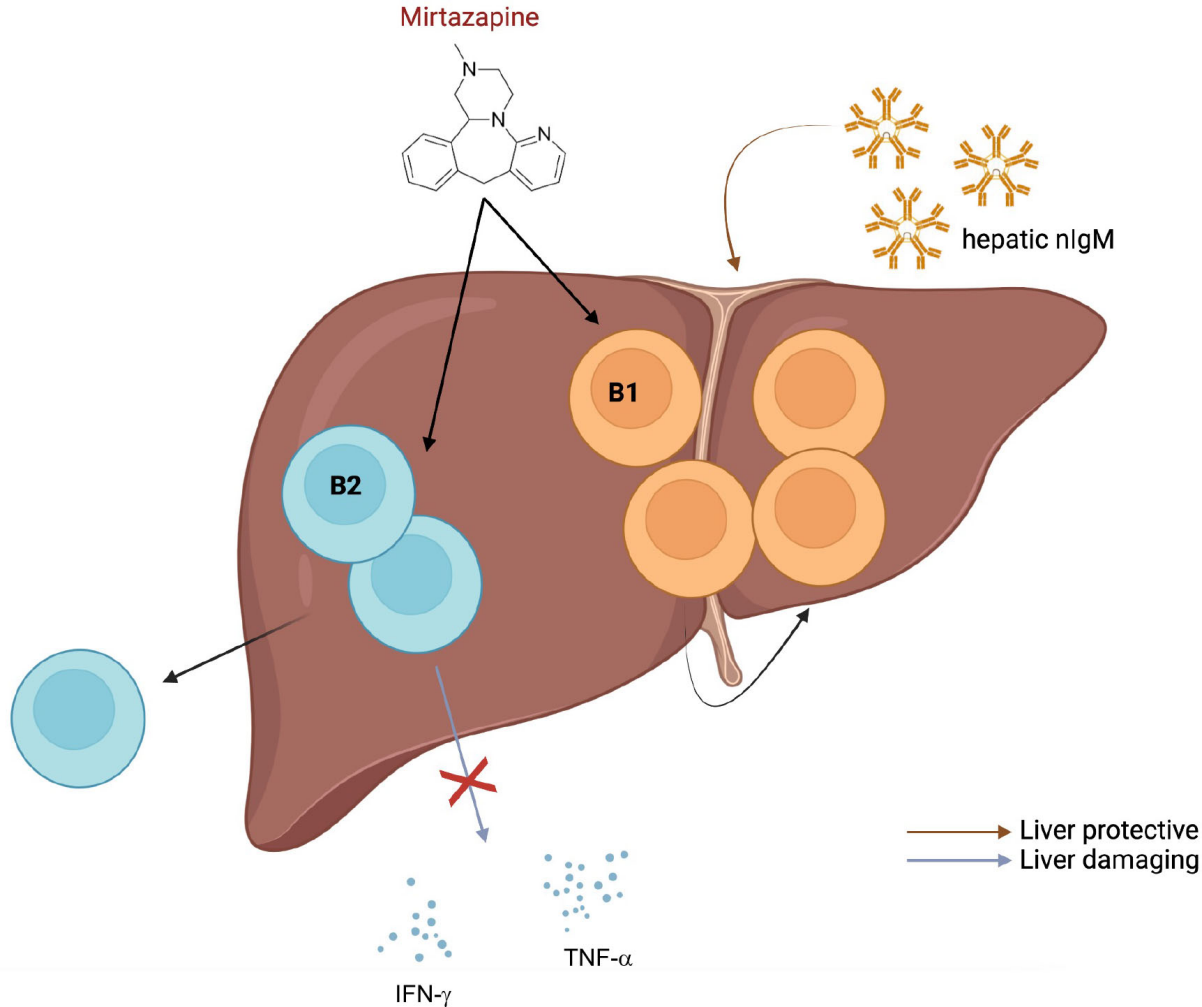
Possible effects of mirtazapine on liver-localized B cells. Almishri and colleagues (44) have demonstrated that treatment of mice with the atypical antidepressant, mirtazapine, leads to an increased proportion of hepatic B1 compared to B2 B cells, suppression of pro-inflammatory cytokine production by B cells and emigration of hepatic B2 B cells from the liver. Figure was created by using biorender.com.

Surprisingly, Almishri et al. (44) showed in a recent study that the atypical antidepressant, mirtazapine, is capable of skewing the B1:B2 B cell ratio in the liver towards B1 B cells and suppressing pro-inflammatory cytokine secretion (**Figure 1**). Consequently, it is conceivable that mirtazapine exerts a liver-protective effect in mice. Since several studies have demonstrated beneficial effects of hepatic nIgM for liver health, it will be interesting to examine effects of mirtazapine treatment on hepatic nIgM repertoire (specificities) and production (quantity). The antidepressant might result in retention of B1 B cells in the liver that produce nIgM favorable for liver regeneration and homeostasis. In general, it is tempting to speculate that the liver may be an important source of nIgM production which is systemically required to balance inflammation. Therefore, altering the hepatic nIgM repertoire might exert beneficial effects on overall health. Elevated cytokine neutralizing hepatic nIgMs together with suppressed production of pro-inflammatory cytokine production post mirtazapine-treatment might have synergistic effects. Furthermore, it will be interesting to determine if effects of mirtazapine are only observed in the liver or also in other organs such as the spleen.

The fact that mirtazapine treatment alters cytokine secretion profiles of hepatic B cells upon stimulation one day post treatment suggests changes in the transcriptome. This is interesting with respect to intracellular signaling and the expression of certain kinases or receptors. Since mirtazapine is known to bind several receptors expressed on the surface of B cells (H1, 5HT3, 5HT3A), elucidating possible pathways might shed light on the regulation of cytokine production in B cells. Mirtazapine-regulated signaling pathways in B cells may crosstalk with other critical pathways such as those downstream of BCR or BAFF-R, a possibility that remains to be elucidated. For instance, H1 is known to signal *via* PLCγ2 (61), an important player of the BCR pathway.

Further, Almishri et al. have demonstrated that mirtazapine treatment leads to increased CXCL10 secretion by monocytes and to increased CXCR3 expression on hepatic B1 B cells retaining this population in the liver. It is possible that mirtazapine also leads to increased expression of other chemo-attractant molecules such as CXCR5 or CXCR4 especially on B2 B cells thus forcing migration to lymph nodes or the spleen (62). This might explain the observed reduction in hepatic B2 B cells post mirtazapine treatment.

In conclusion, given the fact that mirtazapine treatment enriches the liver for B1 B cells and suppresses pro-inflammatory cytokine production it will be interesting to test effects of mirtazapine treatment in AIH and PBC mouse models. In the end, mirtazapine-like drugs might be attractive therapy options in AILD if it can be shown to suppress B cell-derived inflammation and increase beneficial hepatic nIgM titers.

Lastly, since depression is accompanied by a high risk of developing chronic inflammatory diseases (63), mirtazapine treatment might prevent manifestation of inflammation in patients suffering from depression.

## Author contributions

TA and VLJT discussed the concept and wrote the manuscript. All authors contributed to the article and approved the submitted version.
